# The relationship between polymorphisms of *XRCC5* genes with astrocytoma prognosis in the Han Chinese population

**DOI:** 10.18632/oncotarget.13297

**Published:** 2016-11-11

**Authors:** Xue He, Xikai Zhu, Lei Li, Jiayi Zhang, Ruipeng Wu, Yuan Zhang, Longli Kang, Dongya Yuan, Tianbo Jin

**Affiliations:** ^1^ Key Laboratory for Molecular Genetic Mechanisms and Intervention Research on High Altitude Disease of Tibet Autonomous Region, School of Medicine, Xizang Minzu University, Xianyang, Shaanxi 712082, China; ^2^ Key Laboratory for Basic Life Science Research of Tibet Autonomous Region, School of Medicine, Xizang Minzu University, Xianyang, Shaanxi 712082, China; ^3^ Key Laboratory of High Altitude Environment and Gene Related to Disease of Tibet Ministry of Education, School of Medicine, Xizang Minzu University, Xianyang 712082, Shaanxi, China; ^4^ Department of Thoracolumbar Spine Surgery, Second Affiliated Hospital of Inner Mongolia Medical University, Hohhot 010000, China; ^5^ Inner Mongolia Medical University, Hohhot, Inner Mongolia 010050, China; ^6^ Key Laboratory of Resource Biology and Biotechnology in Western China, Ministry of Education, School of Life Sciences, Northwest University, Xi'an 710069, China

**Keywords:** astrocytoma, prognosis, overall survival, single nucleotide polymorphism

## Abstract

**Background:**

Gliomas are highly malignant with a poor prognosis. Studies have reported that DNA repair genes influence risk for glioma, but its relationship with prognosis is unclear. In this study, we want to explore the relationship between DNA repair genes (*XRCC3*, *XRCC4* and *XRCC5*) and prognosis of astrocytoma in the Chinese Han population.

**Materials and Methods:**

160 astrocytoma cases were recruited in our study. Survival probabilities were estimated by using Kaplan–Meier analysis, and significant differences were analyzed by using the log-rank test. Cox proportional hazards models were used to analyze the associations between genotypes with astrocytoma survival. Hazard ratios (HR) and 95% confidence intervals (CI) were estimated using multivariable models. All tests were two-sided and *p* < 0.05 was considered to be significant.

**Results:**

The SNP (rs9288516) in *XRCC5* (HR: 1.69, 95%CI: 1.04 - 2.77, *p* = 0.049), surgical approach (HR: 0.61, 95%CI: 0.43 - 0.88, *p* = 0.003) and chemotherapy (HR: 0.71, 95%CI: 0.50 - 0.99, *p* = 0.029) were associated with astrocytoma prognosis. Further, the “A/A” genotype of rs9288516 in *XRCC5* (HR: 1.67, 95%CI: 1.02 - 2.72, *p* = 0.042) had significantly outcomes after adjusting for potential confounders, patients with poor tumor differentiation and the coexistence of the unfavorable genotypes.

**Conclusion:**

These results suggest that polymorphisms of *XRCC5* play an important role in astrocytoma prognosis in the Chinese Han population which could be used in the determination of astrocytoma prognosis in clinical researches.

## INTRODUCTION

Astrocytoma is a tumor composed of astrocytes, which is the most common type of neural epithelial tumor [1]. Astrocytoma is an invasive growth tumor, the majority of tumor possible recurrence after surgical resection, and the recurrence of the tumor can evolve into anaplastic astrocytoma or pleomorphic glioblastoma. In recent years, the world's major cancer research center has been committed to the study of astrocytoma prognosis of the scholars dedicate themselves to looking for more effective treatment method all around the world. At present, we have no uniform standard about the prognosis and treatment of glioma in domestic and foreign.

The previous literature has recorded the main method to evaluate the prognosis and treatment: 1, CT and other imaging results as a determination of indicators, mainly through a period of time after treatment to determine the change in tumor volume; 2, Survival rate and survival time were used as indicators: survival rate of 1 year, 3 year and 5 year survival rate of tumor patients were observed and survival curves were drawn. The curative effect and prognosis of different treatment groups were observed; 3, the change of the functional status of the patients before and after the treatment was judged [2]. The factors which affect the prognosis of astrocytoma are complicated including age, gender, tumor size, pathological grading, surgical resection, postoperative radiotherapy, KPS (Karnofsky performance Status), chemotherapy and other factors [3-5].

Liu Y, et al. analyzed the effect of NHEJ pathway gene (eg: *XRCC5*, *XRCC4* gene) on glioma in 771 case and 752 controls, found that rs3770502 and rs9288516 in *XRCC5* gene and rs1056503 in *XRCC4* gene increased the risk of glioma [6, 7]. Zhou et al. analyzed the effect of *XRCC3* gene on glioma risk, found that rs861530 and rs3212092 in *XRCC3* gene increased the risk of glioma [8]. But, the relationship between these loci and the prognosis of astrocytoma is not clear. So, we want to determine the effect of DNA repair genes (*XRCC3*, *XRCC4* and *XRCC5*) on the prognosis of astrocytoma in our research.

## RESULTS

### Characteristics of the patients

Specimens from 160 astrocytomas patients (male, 88; female, 72) were available for analysis. 66 cases was less than forty years old and 94 cases were over forty years old in the astrocytomas patients. The pathologic stage distribution was: stage I 18 cases, stage II 78 cases, stage III 64 cases. The 1 and 3 year of the OS of astrocytoma patients were 28.8% and 6.6%, respectively. The characteristic of astrocytomas patients was shown in Tables 1 and 2.

**Table 1:** clinical characteristics of astrocytoma patients

Variety	Classification	Astrocytoma	
		Case(s)	Proportion (%)
Sex	Male	88	55
	Female	72	45
Age	< 40years	66	41.3
	≥40years	94	58.8
WHO classification	WHO I grade	18	11.3
	WHO II grade	78	48.8
	WHO III grade	64	40.0
The extent of surgical resection	Total resection	111	69.4
	Not all	49	30.6
Radiotherapy	Gamma knife	106	66.3
	Conformal radiotherapy	41	25.6
	Not done	13	8.1
	Platinum containing regimens	37	23.1
Chemotherapy	Nimustinecontaining regimens	14	8.8
	Temozolomide containing regimens	7	4.4
	Not done	102	63.8
	Survival	6	3.8
State of survival	Loss to follow-up	7	4.4
	Death	147	91.9
	No progress	6	3.8
State of progress	Progress	152	95.0
	Missing	2	1.3

**Table 2:** Univariate cox regression analysisof prognostic factors for overall survival rates of astrocytoma patients

Variety	Classification	Death cases/Total cases	Median survival time	1 year/3 year overall survival	*p*	HR(95%CI)
Total	/	147/160	11	28.8%/6.6%	/	/
Sex	Male	80/88	11	26.1%/6.8%	0.769	1
	Female	67/72	11	31.9%/6.3%		1.05(0.76-1.44)
Age	< 40years	58/66	11	31.8%/8.6%	0.186	1
	≥40years	89/94	10	26.6%/-		1.23(0.88-1.71)
WHO classification extent of surgical resection	I-II grade	86/96	11	28.1%/	0.612	1
	III grade	61/64	11	29.7%/3.9%		1.08(0.78-1.50)
	Not all resection	98/111	9	16.3%/-	0.003	1
	Total resection	49/49	11	34.2%/9.6%		0.61(0.43-0.88)
Radiotherapy	No	11/14	12	46.2%/-	0.334	1
	Conformal radiotherapy	38/41	9	14.6%/8.8%		1.48(0.75-2.91)
	Gamma knife	99/106	11	32.1%/-		1.18(0.63-2.21)
Chemotherapy	No	96/102	9	23.5%/-	0.029	1
	Yes	51/58	12	37.9%/11.4%		0.71(0.49-0.99)

*p*<0.05 indicates statistical significance; HR: hazard ratios; CI: confidence interval.

### Univariate analysis

As shown in Table 2, we found that better prognosis in astrocytoma patients with total resection (astrocytoma: OS: *p* = 0.003). The 1-year survival of astrocytoma patient with total resection was higher than those with not all resection, respectively. The 1-year survival of astrocytoma patient with total resection was 34.2%, while the 1-year survival of the not all resection astrocytoma patient was 16.3%.

Better prognosis in astrocytoma patients with accept Chemotherapy (OS: *p* = 0.029). The 1-year survival of astrocytoma patient who accept chemotherapy were 37.9%, which were higher than the patient without chemotherapy treatment (1-year survival: 39.4% vs. 23.5%) (Table 2).

No significant association was found between the prognosis of astrocytomas and selected demographic characteristics such as sex, age, WHO classification, and radiotherapy.

**Figure 1:**
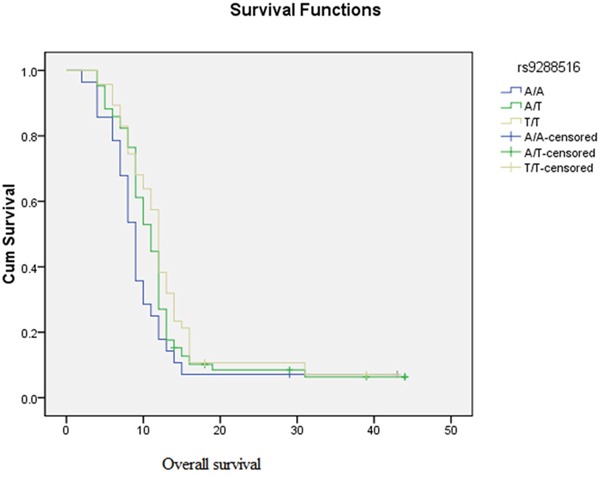
Kaplan–Meier analysis of overall survival is shown for different genotypes of rs9288516 of *XRCC5*

We further explored the role of genetic polymorphisms in the prognosis of astrocytoma, significant association was observed in our study (Table 3). Kaplan-Meier survival curves graphically also emphasize that the “A/A” genotype of rs9288516 in *XRCC5* (X-ray cross-complementing 5) has effect on OS in astrocytomas patients (1-year survival of TT vs. AA: 25% vs.17.9%), which also shorten the astrocytoma patient's survival time, and astrocytoma patients with poor prognosis (HR=1.69) (Figure 1).

**Table 3:** Analysis of the effect of genetic polymorphisms on the overall survival of astrocytoma by Univariate cox regression analysis

Gene	SNP	Genotype	Death cases/Total cases	1,3 year overall survival rate	median survival time	*p*	HR(95%CI)
XRCC5		G/G	99/107	44.9%/5.9%	11	0.597	1
	rs3770502	A/G	46/50	44%/6.7%	10		1.05 (0.74-1.49)
		A/A	2/3	33.3%/-	12		0.55 (0.14-2.22)
		T/T	43/47	25%/-	12	0.049	1
	rs9288516	A/T	78/85	27.1%/6.4%	11		1.2 (0.83-1.75)
		A/A	43/47	17.9%/-	9		1.69 (1.04-2.77)
		G/G	75/83	51.8%/8.6%	12	0.267	1
XRCC4	rs1056503	G/T	55/59	35.6%/4.1%	10		1.29 (0.91-1.82)
		T/T	17/18	44.4%/-	9		1.24 (0.73-2.09)
	rs3212092	C/C	135/148	46.6%/7.2%	11	0.145	1
		T/C	12/12	16.7%/-	9		1.49 (0.83-2.71)
XRCC3		A/A	41/45	51.1%/-	12	0.812	1
	rs861530	A/G	70/78	41%/9.3%	11		1.05 (0.71-1.54)
		G/G	36/37	45.9%/2.7%	11		1.14 (0.73-1.79)

*p*<0.05 indicates statistical significance.

HR: hazard ratios; CI: confidence interval.

### Multivariate analysis

Univariate analysis found that extent of surgical resection and chemotherapy affect the prognosis of astrocytoma patients. Therefore, Multivariate analysis, adjusted for extent of surgical resection and chemotherapy, found that unfavorable genotypes (the “A/A” genotype of rs9288516 in *XRCC5*) had significant outcomes (HR: 1.67, 95% CI: 1.02 - 2.72, *p* = 0.042), which were displayed in Table 4.

**Table 4:** Analysis of the effect of genetic polymorphisms on the prognostic of astrocytoma by Multivariate cox regression analysis

Gene	SNP	Genotype	*p*	HR(95%CI)
XRCC5		G/G	0.629	1
	rs3770502	A/G	0.683	1.08(0.76-1.53)
		A/A	0.401	0.55(0.13-2.24)
		T/T	0.122	1
	rs9288516	T/A	0.22	1.27(0.87-1.84)
		A/A	**0.042**	1.67(1.02-2.72)
		G/G	0.264	1
XRCC4	rs1056503	T/G	0.103	1.34(0.94-1.91)
		T/T	0.593	1.16(0.68-1.96)
	rs3212092	C/C	-	1
		T/C	0.095	1.66(0.92-3.02)
XRCC3		A/A	0.794	1
	rs861530	A/G	0.551	1.13(0.76-1.67)
		G/G	0.552	1.15(0.73-1.80)

*p*<0.05 indicates statistical significance with adjusted for surgical approach and chemotherapy

HR: hazard ratios; CI: confidence interval.

## DISCUSSION

The findings of this study suggested an association surgical approach, chemotherapy, SNP in the *XRCC5* gene and the risk for astrocytoma prognosis in Xi'an population. The results indicated that surgical approach, chemotherapy and *XRCC5* gene influence the prognosis of astrocytoma patients.

### Surgical approach

At present, the majority of scholars had realized that it is the first choice to cure astrocytoma by surgical treatment, which would alleviate the oppression of the surrounding tissue and improve the therapeutic effect. It has been reported that there were significant differences between the total resection and partial resection of the tumor and the length of survival time of malignant glioma is related to tumor resection [9]. The study had confirmed that the 1 and 3 year survival rate of total resection of the tumor (35.92%, 8.9%) was significantly higher than the partial resection of the tumor (17.6%, 1.2%). In our research, we found that better prognosis in astrocytoma patients with total resection. The 1 year survival rates of astrocytoma patient with total resection were 34.2%, while the 1 year survival rates of the not all resection astrocytoma patient was 16.3%. The 1 year survival rates of astrocytoma patient with total resection were higher than those with not all resection, respectively The extent of tumor resection would affect the prognosis, which is one of the independent factors affected the prognosis. The degree of tumor resection is cleaner, the prognosis is better.

### Chemotherapy

Even if surgery has achieved good results in the treatment of glioma, possiblity to recur for most tumors, chemotherapy is one of method to inhibit the progression of astrocytoma, while the treatment outcome was poor. The main reason is the existence of drug resistance and blood brain barrier factors and so on. Although the effect is unsatisfactory, there is still a certain function. In our study, we found that the univariate analysis showed that chemotherapy was statistically significant, 1 and 3year survival rate was significantly higher (39.4%, 12.7%) than patients who did not receive chemotherapy (24.1%, 2.7%). Stewart et al. has used the Meta analysis about the effect of chemotherapy for malignant glioma [10]. The results had showed that patient of glioma received chemotherapy, whose 1 years survival rate increased by 6%; 2 years survival rate of glioblastoma increased by 4%, the average survival time was prolonged for 2 months. So, to a certain extent, astrocytoma patient accepted chemotherapy may have positive role.

### *XRCC5* gene and prognosis of astrocytoma

By multivariate cox regression analysis, we found that rs9288516 in *XRCC5* gene influence the prognosis of astrocytoma patient. Kaplan-Meier curves and log-rank results revealed that astrocytoma patients carrying genotype AA rs9288516 had shorter survival time than those with genotype AT and TT alone. Consistently, astrocytoma patients carrying AA genotype at rs9288516 also had worse survival. It was also reported that *XRCC5* gene polymorphism effect the prognostic of hepatocellular Carcinoma and melanoma, as a risk factor [11, 12].

XRCC5 (X-ray repair cross-complementing gene 5) is one of the double-strand break repair genes, which plays an important role in non-homologous terminal repair. The Ku80 protein was encoded by the XRCC5, and KU70 protein was encoded by the XRCC6 [13]. KU80 protein and KU70 protein forms a heterodimer (ie: KU protein), heterodimer and DNA-PK catalytic subunit (DNA-PKes) composition of DNA-PK [14]. When the XRCC5 gene mutates, the structure of the encoded Ku80 protein may change, it will cause Ku80 protein and Ku70 protein cannot bind or reduce the ability to bind, and will affect the heterodimer formation, so that the ability to mobilize DNA-PKes decreased, DNA-PK cannot play a normal role [15]. The non-homologous end repair function cannot be carried out, which may lead to the occurrence of tumor.

It has been recently reported that a significant increase in XRCC5 expression in hepatocellular carcinoma mice can prevent liver tumors induced by DNA damage [16]. However, the role of the XRCC5 gene in astrocytomas has not been studied. Functional prediction of rs9288516 using software FASTSNP, suggested that rs9288516 might cause a change in the potential binding sites of transcription factors, which may result in instability of XRCC5 messenger RNA transcripts, resulting in dysfunction of XRCC5 expression and ultimately lead to cancer [17].

In our study, we regarded “A/A” of rs9288516 as an increased factor, which was just associated with prognostic effect of astrocytoma in Chinese patients. Compared with the previous studies, we found that *XRCC5* gene is not a risk factor, can be used as a negative factor with prognostic effect of glioma. Our finding suggested that this gene may play a different role in complex diseases. In further studies, we should investigate the different mechanisms of *XRCC5* gene in different diseases. So that it is worth to pay more attention to explore the role of *XRCC5* gene in variety cancers.

Although we have found that the XRCC5 gene may affect the prognosis of astrocytoma patients, there are still some problems that need to be improved in this study. For example, our sample size is relatively small, we should expand the sample in-depth study. Sometimes, individuals may carry risk alleles that will affect the outcome to the extent. So that the interactions effect of gene-environment should be explore in the furture.

### Conclusion

In conclusion, we investigated an association between *XRCC3*, *XRCC4* and *XRCC5* gene polymorphism and the risk and prognosis of astrocytoma in Chinese Han population. We have found evidence suggesting that the genotypes of *XRCC5* may be correlated with increased risk and poor prognosis for astrocytoma. However, the exact mechanism of how the polymorphisms in the related the gene regulate astrocytoma prognosis needs to be further investigated.

## MATERIALS AND METHODS

### Study participants

All astrocytoma patients were Chinese Han who were followed up from December 2010 to April 2014, recruited from the department of Neurosurgery at Tangdu Hospital, Xian City, China. A total of 160 subjects were newly-diagnosed with pathologically verified astrocytoma in this study. Clinical information was collected and regularly updated for the patients with glioma through follow-up and questionnaires. These data include date of age, gender, date of diagnosis of primary tumor, date of surgical resection and surgery extent, treatment with chemotherapy and/or radiotherapy for primary and/or recurrent lesions, date of last follow-up, and status of patient (living/deceased) at the time of last follow-up. We excluded patients with the secondary astrocytoma from a primary cancer and patients received cetuximab at any step as part of their treatment were also excluded.

All participants were informed of the procedures and purpose of the study, and each participant provided signed informed consent forms. The use of samples was approved by the Clinical Research Ethics of Xizang Minzu University and the Tangdu Hospital board.

### Selection of SNPs and methods of genotyping

Five SNPs from 3 genes, previously reported that those gene were associated with glioma, were chosen for analysis in the current study [6-8]. 5 mL of whole blood sample was collected from each individual. DNA was separated from peripheral blood lymphocytes using the Gold Mag-Mini Purification Kit (Gold Mag Co. Ltd. Xian city, China). DNA concentrations were measured using the NanoDrop 2000 (Thermo Scientific, Waltham, Massachusetts, USA). Multiplexed SNP MassEXTENDED assay was designed by SequenomMassARRAY Assay Design 4.0 Software (Sequenom Co. Ltd., San Diego, CA, USA) [18]. SNP genotyping with a standard protocol was performed using SequenomMassARRAY RS1000 (Sequenom Inc., San Diego, CA, USA) [18]. SequenomTyper 4.0 Software (Sequenom Inc., San Diego, California, USA) was used to analyze the data [18, 19].

### Statistical analysis

OS was defined as the time from the date of pathologically confirmed to the date of death or last clinical follow-up. PFS was calculated from the date of the pathologically confirmed to the progression of the disease, death without progression, or last clinical follow-up. We computed basic descriptive statistics for age at diagnosis, gender, WHO grade, extent of surgery, radiation therapy, chemotherapy, and frequencies of five SNPs. Survival distributions were estimated by using the Kaplan-Meier method and difference in the survival was tested using the log-rank test. To estimate the association of the five SNPs with PFS and OS in astrocytoma, the HR and 95% CI were calculated by Univariate Cox proportional hazards model. Multivariate Cox model were performed to compute adjusted HR and 95% CI, after adjusting for potential risk factors. All tests were two-sided and *p* < 0.05 was considered to be significant. All statistics were conducted by SPSS 17.0 (SPSS, Chicago, IL, USA).
